# Ballet as a movement-based contemplative practice? Implications for neuroscientific studies

**DOI:** 10.3389/fnhum.2014.00513

**Published:** 2014-07-17

**Authors:** Marieke K. van Vugt

**Affiliations:** Department of Artificial Intelligence and Cognitive Engineering, University of GroningenGroningen, Netherlands

**Keywords:** yoga, mindfulness, meditation, metacognition, emotion, interoception

There is a rising scientific interest in the neuroscience behind contemplative practices (see e.g., Vago and Silbersweig, 2012 for a review), including movement-based practices such as yoga and tai chi. Given that, it becomes important to ask how such contemplative practices differ from Western movement practices such as dance. In both dance training and contemplative movement, one learns to control the body very precisely, and this requires an assortment of mental skills as well. As a practitioner of both classical ballet and contemplation, and as a neuroscientist who studies contemplation, I will examine how the neural and mental causes and consequences of movement training differ between dance and contemplation. Ballet, rather than modern dance, serves as a good contrast for contemplative practice, because modern dance itself has been influenced substantially by contemplative practice (Hay, 2000). I will compare classical ballet and movement-based contemplative practice on the dimensions of (i) cultivation of attention, (ii) development of interoception, (iii) cultivation of meta-cognition, and (iv) emotion regulation. To date, there are limited studies of movement-based practices, for the obvious reason that movement tends to create artifacts in neuroimaging and EEG measures (e.g., Gwin et al., 2010). I will point out important gaps in our neuroscientific understanding of these phenomena. The results have implications for how we conduct studies of contemplative practitioners and dancers.

## Cultivation of attention

The first thing that happens when engaging in contemplative practice is the cultivation of attention. There is a rich literature on how contemplative practice trains sustained attention (MacLean et al., 2010), attentional stability (e.g., Lutz et al., 2009; van Vugt and Jha, 2011), the ability to deal with conflicting information (Jha et al., 2007), and so on. While most of the research on attention has been done on sitting meditation, embodied practices such as tai chi have been shown to affect attention as well (e.g., Kerr et al., 2008). These behavioral effects are accompanied by specific changes in the brain as well: for example meditation has been associated with an increase in phase-locking to presented stimuli, thought to reflect more selective attention (Lutz et al., 2009), and increased modulation of 7–14 Hz alpha oscillations thought to reflect the direction of attention to relevant inputs (Kerr et al., 2011).

The training in ballet too requires substantial attentional focus, since it involves sustained attention to the details of muscle tension and location of body parts, situational awareness of where the other dancers are, and memorization of movement sequences. Cultivation of focused attention is generally reflected in a strengthening of the fronto-parietal attention network that consists of most importantly the intraparietal sulcus, medial frontal gyrus, inferior frontal gyrus, and anterior cingulate cortex (Corbetta and Shulman, 2002; Dosenbach et al., 2007). It would therefore be interesting to compare the developmental trajectory of fronto-parietal attention network use over the course of dance and contemplative training, which both require thousands of dedicated hours of practice (Slagter et al., 2011). In fact, changes in dedicated neural circuits are already slowly starting to be uncovered (see e.g., Lazar et al., 2005; Hänggi et al., 2010 for reports of brain structure changes in meditators and dancers, respectively).

## Interoception

There has been some controversy on whether contemplation helps to improve interoception. Interoception refers to the ability to feel your body. It is measured in tasks such as the counting of heartbeats. It may not be improved very much by contemplative practices that emphasize sitting still (Khalsa et al., 2008; Sze et al., 2010; Daubenmier et al., 2013), but there is some evidence that it does improve in practitioners of tai chi (Kerr et al., 2008). This is interesting, because improvements in awareness of the outer body have been associated with improvements in motor control (e.g., Wong et al., 2012). Moreover, long-term meditation practice has been associated with, among other things, increased cortical thickness in the right anterior insula (Lazar et al., 2005), a brain structure crucial for interoception (Critchley et al., 2004) (note that this was found in a group of Vipassana meditators, who focus a lot on sensations of breathing in their practice).

Based on these findings, one could imagine that ballet dancers, who are preoccupied with their body all day, have a good sense of interoception. However, an equally likely hypothesis is that dancer's interoception is not different from the general public, since ballet dancers judge the shape of most of their movement in mirrors and are trained to ignore pain signals from their bodies since “the show must go on.” (McEwen and Young, 2011). Many ballet dancers even feel the need to resort to yoga, gyrokinesis, or pilates to attune themselves to their bodies again. In contrast, contemporary dancers typically work without mirrors, and focus more on interoceptive signals.

Consequently, I predict that there is a clear difference between dance forms in the extent to which interoception is developed. Since in classical ballet, dancers tend to rely more on mirrors to correct their position and create the desired shapes with their body, I predict classical dancers have an interoceptive accuracy not different from non-dancers, while for modern dancers, who tend to rely less on mirrors, there should be superior interoceptive accuracy compared to non-dancers. One study that investigated the relationship between interoceptive accuracy (in that case, for emotions) in both dancers and meditators showed that meditators were the most accurate, followed by dancers, who were still more accurate than participants from the general population (Sze et al., 2010). However, this study did not separate modern from classical dancers. Studies of the neural circuitry underlying interoception in dancers are missing altogether. So, even though interoception is likely to be trained in both dance and contemplation, the jury is still out.

## Meta-cognition

A third mechanism that is crucial for both dance and contemplation is meta-cognition. Meta-cognition could be defined as a process of re-describing its knowledge to yourself and comparing it to predictions you have made (Timmermans et al., 2012). In other words, meta-cognition involves observing your own thoughts. Meta-cognition is crucial for monitoring of whether your cognitive processes are developing along the lines you intend to (for example: are you paying attention to that letter on the screen, or are you day-dreaming about what to have for lunch?), and if necessary adjusting them. For ballet dancers, meta-cognition is necessary for checking whether their attention is divided appropriately between monitoring the use of the correct muscles, monitoring the location of fellow dancers, thinking ahead in the memorized movement sequence, and often also creeping in the skin of the character or emotion they are trying to convey.

In contemplation too, meta-cognition is crucial (Kuan, 2012). Meta-cognition helps the yogi to check the alignment of their body, and observe their thoughts and emotions as those unfold, making sure not to get caught up in them. Rather than simply mindlessly performing movements, there is an emphasis on observing how movement affects the body and mind. In addition, meta-cognition is necessary to observe the appropriate balance between tensing and relaxing (e.g., Wallace, 2008). While meta-cognition has not been specifically investigated in the context of movement-based practices, it has in general been associated with BOLD activity in the dorso-medial prefrontal cortex and the anterior insula (Schilbach et al., 2012). In more cognitive tasks, meta-cognition appears to rely more on lateral prefrontal cortex and anterior cingulate cortex (Fox and Christoff, 2014). This is consistent with the idea that a crucial determinant of meta-cognition is working memory capacity. Working memory strategies are what allows the meta-cognitive processes to maintain multiple pieces of information simultaneously and to transform those for alternative needs, e.g., simultaneously maintaining the location of fellow dancers with the memorized sequence of steps.

Interestingly, while ballet dancers tend to use meta-cognition to ensure that they are still delivering the correct product (dance) to the audience, contemplatives use meta-cognition to primarily observe their own mental state. The meta-cognition that dancers use, in view of a project delivery is typically highly critical and judgmental, while yogis train to observe their mind and body with a non-judgmental attitude. It could even be said that an important function of meta-cognition in contemplation is monitoring whether or not one is reacting judgmentally. Consequently, comparing dancers and contemplatives on meta-cognition would be an interesting way to isolate externally-directed from internally-directed meta-cognition. Moreover, it will be interesting to see whether those two types of meta-cognition are associated with different neural correlates, along the lines of the suggested difference between meta-cognition of emotions and bodily state versus cognitive states (Fox and Christoff, 2014).

## Emotion regulation

Emotion regulation is an umbrella term for many processes that serve to influence emotions and align them with ones goals (Todd et al., 2012). It includes reappraisal of events, suppression of emotional reactions, avoidance, coping, and more. An important part of training in many contemplative practices, including movement-based contemplative practices involves techniques of dealing with emotions that could be classified as emotion regulation. Specifically, practitioners cultivate the ability to observe emotions but withhold automatic elaboration and action tendencies that flow from these emotions. Many contemplative practices also have an element of dealing with emotions by refocusing attention on a different stimulus, such as the breath or physical sensations. Not surprisingly then, yoga has been used in the treatment of war veterans with Post-Traumatic Stress Disorder (da Silva et al., 2009). Some regions that are important for emotion regulation, the lateral prefrontal cortex and orbitofrontal cortex, have been shown to have larger activity and density in meditators (e.g., Farb et al., 2007; Hölzel et al., 2008; Grant et al., 2010). These areas are thought to be engaged in emotion regulation by modulating activity in areas such as the amygdala, striatum, and hippocampus (see Vago and Silbersweig, 2012 for a discussion).

Yet I argue that ballet training too, involves some cultivation of emotion regulation skills. Many professional and non-professional dancers describe their dancing as a way to express their emotions, as a kind of catharsis, which is in fact controlled enhancement of emotions (Ochsner et al., 2004). Not surprisingly, many types of dance therapy have been developed to deal with emotional turmoil (e.g., Betty, 2013). I predict that dance therapy and sustained training in dance modulate the connectivity between lateral prefrontal cortex and orbitofrontal cortex on the one hand and the amygdala and hippocampus on the other.

On the other hand, we should also note a crucial difference in the way emotions are experienced in ballet versus contemplative movement practices. While in dance, particularly in theatrical performance, the goal is to experience emotions to the fullest, in contemplative practices emotions are seen as merely an expression of the mind that arises but also disappears again. One could say, in dance emotions are magnified and objectified, while in contemplative practice, the fleeting nature of emotions is emphasized, which actually makes them decrease in magnitude and importance. Recently, it has been suggested that the “stickiness” of emotions is accompanied by a prolonged response in the amygdala (Schuyler et al., 2014). If this is true, this amygdala response should be increased in dancers relative to contemplative practitioners (but note that more regions than just the amygdala are involved in producing the full-blown experience of emotions; Anderson and Phelps, 2002). So, while professional dancers and contemplatives both work on adapting and regulating their emotions, they do this in different ways.

## Synthesis and future directions

I have shown how there are many commonalities between classical ballet and contemplative movement practices, in the domains of attention training, interoception, meta-cognition and emotion regulation. Yet, ballet training and contemplation also differ in the presence of a non-judgmental attitude and importance given to emotions.

While the behavioral correlates are relatively well-described, the neural pathways remain under-explored. Furthermore, these commonalities and differences between dance and contemplative movement practices could have implications for the studies we do. For example, using ballet dancers as a control group for long-term contemplative practitioners could be very interesting, because both populations have developed a strong focus on their craft over a life-time of concentrated practice, and developed skills in the domain of attention, meta-cognition, interoception, and emotion regulation. In contrast, the groups differ in their non-judgmental attitude and solidity ascribed to emotions. Comparing these groups therefore allows one to tease apart the judgmental and non-judgmental modes of attention, meta-cognition, and emotion regulation, and could thereby help define its neural and behavioral correlates.

In addition, it would be interesting to engage dancers and practitioners of contemplative movement practices together in neurophenomenological experiments. Neurophenomenology (Varela, 1996; Cosmelli and Thompson, 2007) involves analyses of brain activity informed by disciplined introspection on the part of the participants. Since both of these groups of people have spent considerable time observing and honing their bodies and minds, such experiments could help to elucidate the detailed mechanisms behind these types of training, as well as the time course of the arising of judgmental attitudes.

### Conflict of interest statement

The author declares that the research was conducted in the absence of any commercial or financial relationships that could be construed as a potential conflict of interest.
